# Domain-general and domain-specific cognitive factors mediating the relationship between math anxiety and mathematical performance in primary school children

**DOI:** 10.1038/s41598-025-30898-2

**Published:** 2026-01-14

**Authors:** Elora Taieb, Grégoire Borst, Lorna Le Stanc, Olivier Houdé, Irene Altarelli, Teresa Iuculano

**Affiliations:** 1 CNRS, LaPsyDÉ, Université Paris Cité, Paris, F-75005 France; 2 INSPE, Sorbonne Université, Paris, France; 3 Institut Universitaire de France, Paris, F-75005 France; 4 Department of Systems Medicine, University of Rome Tor Vergata, Via Montpellier 1, Rome, 00133 Italy; 5 Department of General Psychology, University of Padua, Via Venezia 8, Padova, 35131 Italy

**Keywords:** Math anxiety, Mathematical performance, Cognitive mediators, Domain-general skills, Domain-specific skills, Primary school children, Mathematics and computing, Neuroscience, Psychology, Psychology

## Abstract

Math anxiety (MA) has been widely recognized as a barrier to mathematical achievement, yet the cognitive mechanisms underlying its effects on different mathematical outcomes remain poorly understood. This study applies a multiple mediation model to examine how MA affects three key domains of mathematical performance – word problem-solving, calculation, and math fluency – through both domain-general (working memory, inhibition, cognitive flexibility, selective attention) and domain-specific (transcoding, ordering, symbolic and non-symbolic comparison) cognitive factors. A sample of 472 French third-graders (aged 7.7–9.4 years) was analyzed using structural equation modeling (SEM) to identify cognitive mediators of the relationship between MA and mathematical outcomes, while controlling for sex, age, nonverbal IQ, socioeconomic status, and general anxiety. Results revealed that among domain-general factors, only working memory mediated the effects of MA on both word problem-solving and calculation. Among domain-specific factors, symbolic skills significantly mediated the link between MA and both calculation and math fluency abilities, whereas non-symbolic skills did not play a mediating role for any mathematical outcomes. Together, these findings provide novel insights into the interplay between domain-general and domain-specific processes in contributing to the effects of MA on mathematical performance, highlighting potential targets for educational interventions during the early years of formal schooling.

**Supplementary Information:**

The online version contains supplementary material available at 10.1038/s41598-025-30898-2.

Proficiency in mathematics is essential in today’s modern society and has been identified as a key predictor of academic and professional success^1,2^. Math anxiety (MA) – defined as a state of tension, apprehension, or fear during mathematical activities^3^ – poses a significant barrier to mathematical performance. Notably, research has consistently highlighted the pervasive impact of MA on mathematics in both children and adults^4–7^, and across diverse cultural contexts^8^. However, despite substantial evidence of its adverse effects, the mechanisms through which MA disrupts various mathematical abilities remain poorly understood. This is particularly relevant for the early years of formal schooling – a critical learning stage, wherein early difficulties can cascade into pervasive, long-lasting negative educational outcomes^9^.

Prior research in primary school children has demonstrated that MA can affect various mathematical abilities, including word problem-solving^10^, calculation skills^10,11^, and math fluency^12^. Yet, these three mathematical abilities have not been concurrently analyzed within a single cohort, leaving the specific contribution of MA to each mathematical ability unclear. In adults, more holistic approaches have revealed that MA negatively impacts math fluency but not word problem-solving^13,14^. Within this context, findings for calculation skills remain inconsistent, as some studies identified an effect of MA on calculation^14^, whereas others did not^13^. Critically, the use of separate statistical models for each mathematical outcome within these studies has overlooked the covariance between mathematical variables, limiting the possibility to disentangle the unique effects of MA on each of these abilities. Addressing these limitations by simultaneously analyzing diverse mathematical outcomes within an integrated framework is necessary to better understand the cognitive mechanisms linking MA to mathematics achievement. Moreover, applying this approach to a population of primary school children represents a novel contribution, as such investigations have not been conducted within this developmental period.

Thus far, research investigating the links between MA and mathematical performance has predominantly focused on domain-general skills, particularly highlighting the role of working memory as a key mediator^5,15^. This result aligns with the Processing Efficiency Theory^16^, which posits that anxiety – as sometimes experienced in mathematical contexts – may impose an additional cognitive load on working memory resources, subsequently hindering mathematical performance^3^. Evidence also suggests that adults with high MA struggle with inhibiting irrelevant information^17–19^, efficiently shifting between different types of arithmetic problems^20^, and may display an attentional bias toward mathematical content^21^. However, only few studies have comprehensively examined whether different types of domain-general skills – such as working memory, inhibition, cognitive flexibility, and selective attention – could mediate the relationship between MA and mathematical performance. Among these, one study in adults found that working memory was the only executive function that mediated the link between MA and mathematics^22^; while another conducted with adolescents identified cognitive flexibility as a key mediator^23^. Furthermore, research with fourth- and fifth-grade children showed that MA was associated with increased inattention, which negatively impacted working memory and, subsequently, mathematics achievement, suggesting a serial mediation pathway^24^. Yet, none of these studies have integrated all of these domain-general skills into a unified mediation model.

The investigation of domain-specific cognitive skills as potential mediators of the relationship between MA and mathematical performance has been even scarcer. One study in adults found that symbolic number comparison abilities, as well as working memory, mediated the relationship between MA and arithmetic calculation, although other cognitive skills were not examined^25^. In contrast, research with first- to third-grade children reported that the link between MA and mathematics achievement – assessed through a diverse set of mathematical tasks – was not mediated by single-digit numerosity processing, whether symbolic or non-symbolic, but was exclusively mediated by working memory^26^. To date, the mediating role of a broad range of domain-specific skills in the relationship between MA and distinct mathematical outcomes (i.e., word problem-solving, calculation, and math fluency) has not been thoroughly investigated. Critically, other domain-specific skills that may be affected in individuals with high MA, such as number ordering^27–29^ and approximate number system (ANS) acuity^30^, have not been taken into account in previous investigations. Finally, no research has yet examined the potential mediating effect of transcoding skills, despite their established role for the successful development of mathematical abilities^31,32^.

Overall, and to date, no study has comprehensively examined the cognitive mechanisms mediating the link between MA and different mathematical outcomes through an integrated approach that considers both domain-general and domain-specific pathways simultaneously. Furthermore, while evidence suggests that the relationship between MA and mathematical performance may already be shaped by factors such as sex^33,34^ and SES^35^ as early as primary school, these variables have not been systematically taken into account as potential confounders in prior research. Moreover, and consistent with prior recommendations, we also controlled for general anxiety to ensure that the observed associations were specific to MA^10,36^.

The present study aims to address these gaps in the literature by examining, for the first time and within a narrow age-range of third-graders: (i) the impact of MA on three core and distinct areas of mathematics – namely, word problem-solving, calculation, and math fluency; and (ii) the extent to which both domain-general and domain-specific cognitive skills mediate the relationship between MA and these mathematical outcomes. Focusing on third grade is particularly relevant because this developmental and educational stage marks a key shift from basic to more complex mathematical learning^37,38^, as well as a critical period in MA development^10^ – making it an ideal window for understanding its cognitive underpinnings. In a sample of 472 French third-graders, we employed structural equation modeling (SEM) to conduct a multiple mediation analysis that simultaneously includes all these potential cognitive mediators and mathematical outcomes, while accounting for covariances between variables. Importantly, the present study is the first to systematically account for various confounding factors – including sex, age, month within the academic year, nonverbal IQ, SES, and general anxiety – when examining the relationship between MA and mathematics performance. Overall, this approach enables a nuanced examination of whether domain-general, domain-specific, or both types of cognitive skills mediate the link between MA and mathematics during early formal schooling, and whether these pathways vary across different mathematical outcomes.

## Results

### Descriptive statistics

Descriptive statistics for all raw observed variables included in the analyses are summarized in Table 1. Bivariate correlations among the observed variables are reported in the Supplementary Information (Table S1).

**Table 1:** Descriptive statistics for observed variables. Parental education and occupation, reported here for descriptive purposes, were used solely to compute the SES composite score and were not included as individual covariates in the analyses. For the cognitive measures, lower scores on the Inhibition, Cognitive flexibility, and ANS tasks indicate better performance. In the Inhibition task, the scoring procedure adjusts for processing speed via a baseline condition with no interference^39^. Negative scores indicate that a participant performed better in the Inhibition than in the baseline condition – which occurred in three participants out of the whole sample. ANS = approximate number system; SES = socioeconomic status.

Variable	*N*	Mean	SD	Min	Max
**Math anxiety**					
SEMA score	461	39.37	13.53	20	86
**Mathematical achievement**					
Word problems	472	6.45	3.24	0	12
Mental calculation	472	29.66	9.01	0	44
Written calculation	461	12.56	3.01	1	21
Math fluency	472	38.96	13.23	6	86
**Domain-general cognitive skills**					
Digit span backward	472	6.64	1.73	3	12
Digit span ascending	471	6.00	2.03	1	11
Spatial span backward	472	5.37	1.95	0	11
Inhibition	472	33.30	19.42	−17.80	123.80
Cognitive flexibility	470	102.11	40.98	5.20	403.10
Selective attention	471	53.44	13.60	9	107
**Domain-specific cognitive skills**					
Number dictation	461	13.78	2.64	4	16
Number reading	459	14.83	1.85	4	16
Symbolic comparison	461	39.41	8.08	16	56
Ordering	384	15.27	4.49	1	28
Non-symbolic comparison of small quantities	461	38.23	6.41	18	54
ANS acuity (*w*)	458	0.31	0.25	0.02	3.37
**Covariates**					
Sex (% of girls)	472	51.91%			
Age	472	102.61	4.70	92	113
Testing month	472	6.19	3.22	1	11
Nonverbal IQ	472	13.95	3.15	7	24
Parental education	384	13.56	3.79	0	20
Parental occupation	414	45.50	23.14	14.21	88.70
SES composite (z score)	421	0	1	−2.70	1.99
General anxiety (R-CMAS score)	461	11.98	5.99	0	28

### Measurement model (CFA)

Before testing the SEM mediation model, a confirmatory factor analysis (CFA) was conducted to evaluate the measurement model and verify that the latent variables adequately represented their observed indicators^40^. All observed variables (i.e., indicators) showed high and significant factor loadings, confirming that latent variables reliably captured their respective indicators (Table 2). Fit indices for the measurement model indicated a good fit to the data (χ^2^(11) = 14.283, *p* =.218, CFI = 0.997, RMSEA = 0.024, SRMR = 0.019)^41^.

**Table 2:** Loadings of indicators (observed variables) on latent variables. All coefficients are standardized coefficients and are significant at *p* <.001. SE = standard error.

Latent variable	Indicator	β	SE
Calculation	Mental calculation	0.87	0.05
	Written calculation	0.76	0.05
Working memory	Digit span backward	0.56	0.05
	Digit span ascending	0.60	0.05
	Spatial span backward	0.57	0.05
Transcoding	Number dictation	0.77	0.07
	Number reading	0.72	0.08

### Structural equation model (SEM)

**Fig. 1:**
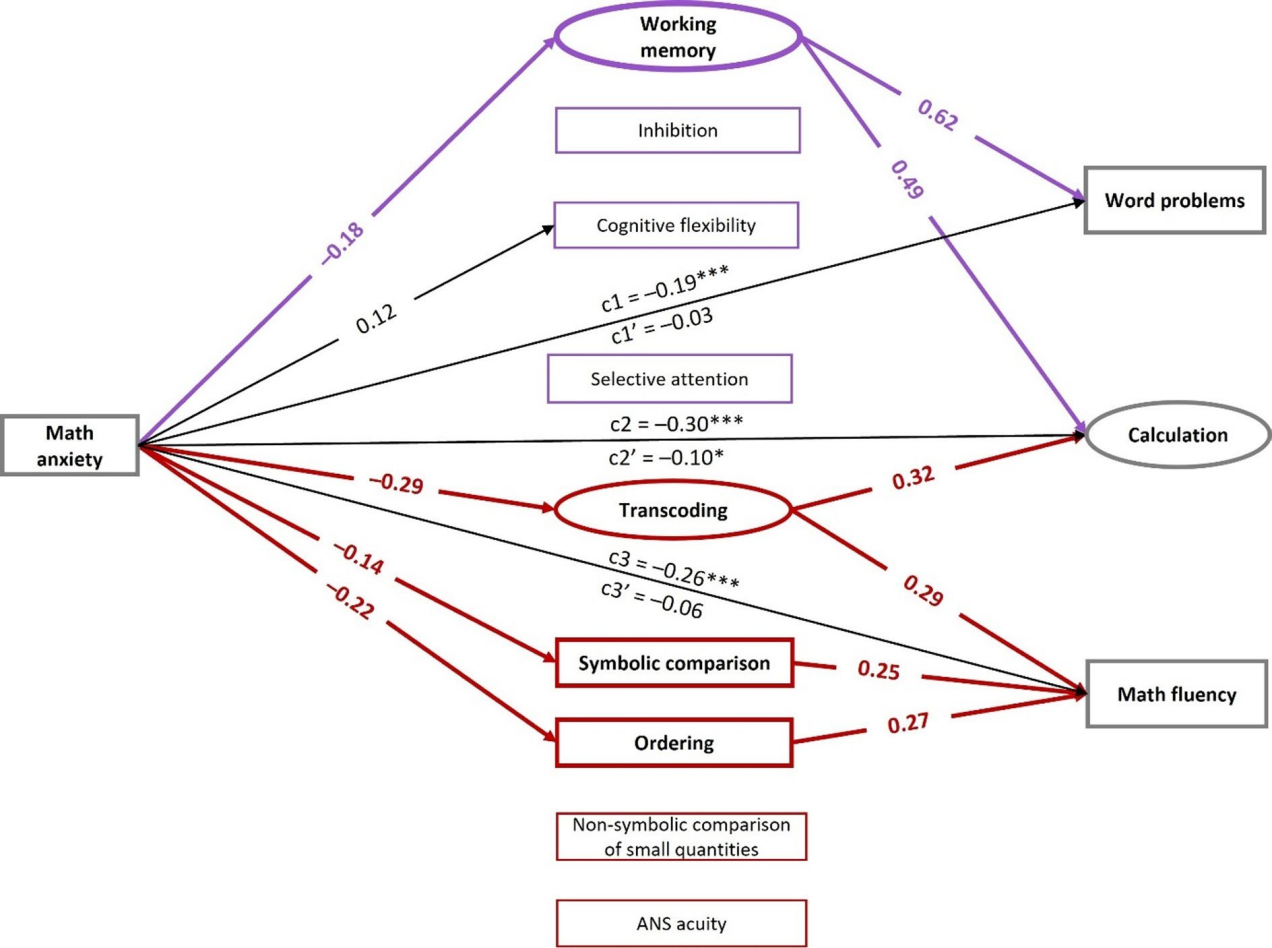
SEM mediation model with total, direct and indirect effects of MA on mathematical outcomes. All coefficients are standardized coefficients. Latent variables are depicted by ovals and observed variables by squares. Domain-general factors are represented in purple and domain-specific factors in red. For ease of presentation, covariances, nonsignificant paths, and covariates – including sex, age, month within the academic year, nonverbal IQ, SES, and general anxiety – are not shown. Total and direct effects are labeled c and c’, respectively. Significant mediation effects are bolded. ANS = approximate number system. *p <.05. ***p <.001.

The SEM analysis assessing the influence of MA on the three mathematical outcomes of interest – namely, word problem-solving, calculation, and math fluency – through the tested cognitive variables exhibited a good fit to the data (χ²(75) = 106.627, *p* =.010, CFI = 0.990, RMSEA = 0.029, SRMR = 0.017)^41^. The results of the model are depicted in Fig. 1. Comprehensive details regarding all path coefficients, as well as variance and covariance estimates, are available in Supplementary Table S2.

#### Bivariate associations

The results revealed that the total effect of MA on mathematical outcomes was significant for all three math abilities, including word problems (path c1: β = − 0.19, *p* <.001), calculation (path c2: β = − 0.30, *p* <.001), and math fluency (path c3: β = − 0.26, *p* <.001). Among domain-general cognitive skills, MA significantly influenced working memory (β = − 0.18, *p* =.005) and cognitive flexibility (β = 0.12, *p* =.030), whereas no significant effects were observed for inhibition (β = − 0.01, *p* =.853) and selective attention (β = − 0.03, *p* =.576). Regarding domain-specific cognitive skills, significant relationships were identified between MA and symbolic skills, namely transcoding (β = − 0.29, *p* <.001), symbolic comparison (β = − 0.14, *p* =.007), and number ordering (β = − 0.22, *p* <.001). No significant effects were found for non-symbolic skills, including ANS acuity (β = 0.10, *p* =.062) and non-symbolic comparison of small quantities (β = − 0.02, *p* =.711). In relation to cognitive predictors of mathematical performance, both domain-general and domain-specific skills emerged as significant contributors. Specifically, working memory predicted word problem-solving (β = 0.62, *p* <.001) as well as calculation abilities (β = 0.49, *p* =.001). Transcoding skills were associated with calculation abilities (β = 0.32, *p* =.001) and math fluency (β = 0.29, *p* <.001). Lastly, symbolic comparison (β = 0.25, *p* <.001) and number ordering (β = 0.27, *p* <.001) skills predicted math fluency.

#### Mediation pathways

The analysis of indirect effects (Table 3) showed that the relationship between MA and word problem-solving was significantly mediated by working memory. Additionally, both working memory and transcoding skills emerged as significant mediators of the link between MA and calculation abilities. The association between MA and math fluency was significantly mediated by symbolic domain-specific cognitive skills including: transcoding, symbolic comparison, and number ordering skills. Notably, the direct effects of MA on word problem-solving (path c1’: β = − 0.03, *p* =.442) and math fluency (path c3’: β = − 0.06, *p* =.139) were no longer significant after accounting for the mediating variables, providing evidence of full mediation effects^42^. The direct effect of MA on calculation (path c2’: β = − 0.10, *p* =.022) remained significant, indicating a partial mediation effect for this outcome.

**Table 3:** Indirect effects of the SEM multiple mediation model. All coefficients reported are standardized coefficients. Significant mediation effects are bolded. CI = confidence interval; MA = math anxiety; ANS = approximate number system.

Mediation paths	Word problems		Calculation		Math fluency	
	β	95% Boot CI	β	95% Boot CI	β	95% Boot CI
MA → Working memory → Math	**–0.11**	**[–0.27**,** − 0.03]**	**–0.09**	**[–0.21**,** − 0.02]**	–0.03	[–0.09, 0.01]
MA → Inhibition → Math	–0.00	[–0.01, 0.01]	–0.00	[–0.01, 0.01]	–0.00	[–0.01, 0.01]
MA → Flexibility → Math	–0.00	[–0.01, 0.02]	–0.00	[–0.02, 0.01]	0.00	[–0.01, 0.02]
MA → Selective attention → Math	0.00	[–0.01, 0.01]	0.00	[–0.01, 0.01]	–0.00	[–0.01, 0.01]
MA → Transcoding → Math	–0.05	[–0.11, 0.03]	**–0.09**	**[–0.18**,** − 0.02]**	**–0.09**	**[–0.16**,** − 0.03]**
MA → Symbolic comparison → Math	0.00	[–0.02, 0.03]	–0.01	[–0.03, 0.02]	**–0.04**	**[–0.07**,** − 0.01]**
MA → Ordering → Math	–0.00	[–0.04, 0.06]	–0.01	[–0.04, 0.05]	**–0.06**	**[–0.10**,** − 0.02]**
MA → Non-symbolic comparison of small quantities → Math	–0.00	[–0.01, 0.01]	–0.00	[–0.02, 0.01]	0.00	[–0.01, 0.01]
MA → ANS acuity → Math	0.00	[–0.01, 0.02]	–0.00	[–0.02, 0.01]	0.00	[–0.01, 0.01]

## Discussion

The present study aimed at: (i) assessing the effects of MA on three core and distinct mathematical abilities, namely word problem-solving, calculation, and math fluency; and (ii) examining the mediating role of both domain-general and domain-specific cognitive skills in the relation between MA and those mathematical outcomes. Importantly, the present investigation was conducted within a narrowly defined developmental window, as participants were all third-graders, thereby limiting developmental variability and providing a unique snapshot onto the aforementioned relationships during a critical stage of formal math learning^38^ and development of MA^10^. To address the present objectives, a comprehensive multiple mediation model was constructed using a SEM approach. This model examined the indirect contributions of cognitive skills to all three mathematical outcomes considered simultaneously, while controlling for key covariates, including sex, age, month within the academic year, nonverbal IQ, SES, and general anxiety.

The findings revealed that MA had a detrimental effect on each of the three mathematical outcomes considered – i.e., word problems, calculation, and math fluency. Furthermore, our mediation analysis highlighted that both domain-general and domain-specific cognitive skills mediated the relationship between MA and mathematical outcomes. Notably, specific mediators varied by mathematical outcome. Namely: working memory mediated the link between MA and word problem-solving; while transcoding, symbolic comparison, and ordering skills mediated the relationship between MA and math fluency. In relation to calculation, both working memory and transcoding skills emerged as significant mediators of its link to MA. No mediation effects were identified for non-symbolic skills on the link between MA and mathematics.

Concerning the influence of domain-general cognitive skills on the MA-mathematics relationship, the present results are in line with previous meta-analyses identifying working memory as a key mediator of the link between MA and mathematical performance^5,15^. This mediation effect, observed exclusively for higher-order mathematical abilities such as calculation and word problem-solving, supports the Processing Efficiency Theory^16^. This theory posits that anxiety experienced during cognitively demanding tasks can induce cognitive overload, thereby depleting working memory resources and impairing performance. Consistent with this theoretical framework, working memory acted as a mediator in the relationship between MA and mathematics only for mathematical tasks that place high demands on working memory^3,43^ – i.e., calculation and word problem-solving – but not for math fluency, which primarily relies on retrieval of arithmetic facts from long-term memory, at least in typically developing children of this age^44^. Notably, other domain-general skills, such as inhibition, cognitive flexibility, and selective attention, did not emerge as mediators of the relationship between MA and mathematical outcomes. This may, in part, reflect the prominence of working memory among several domain-general skills in predicting mathematics achievement at this stage of development^45^. In fact, only working memory was significantly related to mathematical performance – particularly in word problems and calculation – whereas other domain-general skills were not (see Fig. 1 and Supplementary Table S2). Inhibition and cognitive flexibility may nonetheless become more critical in later stages of development, particularly during advanced mathematics learning which requires adopting adaptive strategies and overcoming potential biases induced by prior knowledge^46,47^. Such developmental dynamics could account for the mediating role of cognitive flexibility – but not working memory – observed in adolescents aged 11 to 14 in a previous study^23^. However, this contrasting result may also stem from task-related differences, as the mathematics assessment in that study included an inference task which may require a greater involvement of cognitive flexibility^23^. In line with this, the absence of a relationship between MA and inhibition in the present study may also reflect task characteristics, as our inhibition measure^48^ involved non-numerical stimuli. For instance, prior studies in adults have reported that inhibitory difficulties in math-anxious individuals may emerge only when responding to numerical stimuli^17^, or may be more pronounced for numerical than for non-numerical stimuli^49^. In addition, differences in the use of numerical versus non-numerical stimuli may help clarify the lack of observed contributions of inhibition to mathematical performance (Fig. 1, Table S2), in line with previous findings indicating that associations between inhibition and mathematics achievement tend to appear only when inhibition tasks incorporate numerical material^50^. Future studies should thus systematically compare executive function tasks involving numerical and non-numerical stimuli to provide a more fine-grained understanding of the links between executive functions, MA, and mathematics outcomes.

In relation to the influence of domain-specific cognitive skills on the link between MA and mathematical outcomes, the present findings reveal that all symbolic skills assessed – i.e., transcoding, symbolic comparison, and number ordering – served as mediators, depending on the specific mathematical outcome considered. Critically, no such role was observed for non-symbolic skills. This pattern is consistent with previous studies in adults suggesting that MA primarily affects symbolic rather than non-symbolic processes^51–53^. One potential explanation for this is that high levels of MA may elicit fear and aversion toward mathematical symbols, thereby impairing performance on symbol-dependent tasks only. In contrast, non-symbolic processing seems to be less prone to such emotional interference^4,13^. Importantly, the mediating role of symbolic processing skills in the relationship between MA and mathematics emerged only for mathematical tasks uniquely involving Arabic digit stimuli, such as math fluency and arithmetic calculation, and not for word problem-solving. This finding aligns with previous research^25^ and underlines the idea that basic number processing mainly supports these aspects of arithmetic, while word problem-solving relies more strongly on working memory resources^25,54^.

Overall, our results indicate that the impact of MA on mathematical performance is mediated by domain-general (i.e., working memory) as well as domain-specific (i.e., symbolic processing) cognitive factors. This corroborates findings from adult populations observing similar mediation effects^25^ and further extends them by showing that these effects remain robust even when various other domain-general and domain-specific cognitive factors are considered simultaneously. However, our results diverge from those of a previous study in primary school children, which found that while working memory mediated the relationship between MA and mathematics, domain-specific factors such as symbolic comparison did not^26^. One possible explanation for this discrepancy may lie in the mathematical measures employed in that study, which did not distinguish between distinct mathematical outcomes (i.e., word problems, calculation, and math fluency)^26^, thus potentially masking the nuanced contributions of domain-specific factors.

The present study advances our understanding of how MA influences different aspects of mathematical performance during a critical phase of formal math learning^38^ and development of MA^10^. These findings highlight that the cognitive pathways through which MA impacts mathematical performance are already evident by third grade, underlining the need for early intervention to prevent long-term negative academic and professional outcomes^55^. The present results also suggest that targeted cognitive interventions aimed at enhancing working memory and symbolic skills could help mitigate the adverse effects of MA on mathematics achievement^56^. For instance, a combined training in working memory and symbolic skills has been shown to significantly improve mathematical performance in children aged 5 to 7^57^. Although this study did not assess MA, it raises the possibility that strengthening such domain-general and domain-specific cognitive skills may indirectly counteract the detrimental effects of MA on mathematical performance.

The present study has some limitations that should be acknowledged. First and foremost, the non-longitudinal nature of our design precludes any causal interpretation of the relationships observed. Longitudinal studies will be necessary to clarify the causal pathways between MA and subsequent mathematical performance. Additionally, the debated directionality of the relationship between MA and mathematics (i.e., whether MA influences mathematics or vice versa)^58^ highlights the importance of considering potential reverse pathways as well. While our study was not designed to robustly examine bidirectional effects statistically, future research should investigate whether children with lower domain-general or domain-specific skills are more likely to develop poorer mathematical proficiency and, subsequently, higher levels of MA. In this sense, intervention studies may also serve as a valuable means to clarify the causal nature of these relationships and to determine whether targeted training of working memory and symbolic skills can effectively alleviate the detrimental impact of MA on mathematical performance in primary school children. Beyond longitudinal and interventional approaches, experimental paradigms that manipulate children’s emotional states^59^ could also shed light on the causal impact of MA on cognitive processes and mathematical performance.

Furthermore, although restricting the sample to third-graders constituted a strength of the present design – as it provided a tight developmental window on how MA relates to mathematics during a key transition period in primary school^10,37,38^ – it may also limit the generalizability of the findings in relation to potential developmental changes in the cognitive pathways linking MA to mathematics across different age-groups. Future cross-sectional or longitudinal studies including multiple age-groups (or time-points) will be necessary to determine how these relationships may evolve across broader developmental spans.

Moreover, even though our mathematical assessment covered a broad range of abilities, future research should consider complementing our findings using other types of arithmetical problem-solving protocols and/or think-aloud procedures^60,61^, to capture children’s problem-solving strategies and provide a more detailed account of the mechanisms linking MA to mathematics. Future studies should also systematically examine how presentation formats (e.g., oral problem-solving versus written problem-solving) may influence task performance and enhance reliability by including both oral and written formats across different mathematical outcomes, as our study focused primarily on the main presentation formats appropriate for this age range^62,63^. Additionally, future research should examine in more detail whether distinct subcomponents of working memory (e.g., storage component, manipulation component)^64^, as well as other executive functions (e.g., interference control, response inhibition)^65^, may differentially contribute to the link between MA and mathematics. Lastly, it is important to note that the present study focused exclusively on cognitive mediators, leaving other potentially influential factors unexplored. Notably, the relationship between MA and calculation was only partially mediated by our cognitive measures (namely, working memory and transcoding). This suggests that other factors, not explored here, may be contributing to this relation. For example, emotional factors such as mathematics self-concept^66^ and environmental influences – e.g., participating in mathematical extracurricular activities^36^ – are also likely to mediate the link between MA and mathematics achievement. Addressing these gaps in future research would provide a more holistic understanding of the multifaceted relationship between MA and mathematical outcomes.

In conclusion, the present study advances our understanding of the underpinnings of the link between MA and mathematics achievement in primary school children by revealing the pervasive influence of MA on a range of critical mathematical abilities, encompassing word problem-solving, calculation, and math fluency. Crucially, these results underline the mediating roles of both domain-general skills – particularly working memory – and domain-specific skills – including symbolic processing abilities – in the relationship between MA and mathematics. Altogether, these findings highlight the potential of targeted interventions aiming at enhancing these cognitive skills to support children experiencing high levels of MA during the early years of formal schooling.

## Methods

### Participants

This research was conducted as part of a broader project exploring the environmental, cognitive, and affective determinants of mathematics and reading achievement among primary school children. Participants were third-graders enrolled in 19 elementary schools across socioeconomically diverse areas in and around Paris, France. Informed written consent was obtained from the parents of 597 children prior to their participation. Yet, data were analyzed only for children meeting the following inclusion criteria: normal or corrected-to-normal vision; absence of auditory, neurological, or neurodevelopmental disorders; a nonverbal IQ within 2 standard deviations or higher relative to the age norm; no history of grade retention or acceleration; and acquisition of the French language before the age of three. These criteria were intended to ensure a representative sample of typically developing children and to exclude cases with potential academic or linguistic difficulties that could confound task performance. After applying these criteria, the final sample comprised 472 participants (245 girls; *M*_age_ = 102.6 months; *SD* = 4.7; range = 92–113 months).

### Procedure

Data collection for this study took place from May 2022 to December 2023. Since children were tested at varying times throughout the academic year, schooling effects possibly unrelated to age^67^ were accounted for by including the month of testing as a covariate of no interest in the analyses. Trained research assistants conducted individual testing sessions with each child in a quiet room within their school. Before participation, each child provided oral consent to take part in the respective testing sessions. The assessment spanned four sessions, each lasting approximately 30 min, and separated by an interval of one week on average. Specifically, with regard to the present study, mathematical outcomes were measured in session 1, domain-general cognitive skills in session 2, and domain-specific cognitive skills alongside MA and general anxiety in session 4. Session 3 included additional assessments of reading-related cognitive factors, which are not part of the present study. Additionally, parental questionnaires provided demographic and SES data. Ethical approval for the study was granted by the local ethics committee of Université Paris Cité (N° IRB: 00012023-42). The study was conducted in accordance with relevant national and international regulations governing research involving human participants.

### Measures

#### Math anxiety (MA)

MA was assessed using the Scale for Early Mathematics Anxiety (SEMA)^10^, adapted into French (see Supplementary Table S3). Since a validated French version was not previously available, the questionnaire was translated by a bilingual laboratory member and subsequently reviewed by the first author, a native French speaker, to ensure the translation was clear and easily understandable. The SEMA is a self-report questionnaire designed for use with primary school children. It comprises a total of 20 items depicting situations involving mathematics (e.g., solving addition problems; doing math homework). Items were read aloud to participants by the experimenter, while concurrently being displayed on a sheet of paper in front of the child. Children were asked to indicate how nervous they would feel in each given situation by using a 5-point Likert scale, illustrated with smiley faces, ranging from 1 (*not nervous at all*) to 5 (*very very nervous*). Two practice items unrelated to situations involving mathematics were administered beforehand to confirm understanding of the procedure and the task. The MA score was computed by summing responses across all items. Internal consistency of the scale was confirmed in the current sample with a Cronbach’s α of 0.87, which was identical to the reliability of the original scale in English^10^.

#### Mathematics achievement

**Word problem-solving abilities** were evaluated using the *Word Problems subtest* from the ZAREKI-R battery^68^. In this subtest, a total of six word problems are presented orally by the examiner, and children are required to answer each problem verbally, without a time limit. Scoring criteria assign two points if the child gives the correct response without requesting the problem to be repeated; one point if the correct response is given after the examiner repeated the problem once; and zero points for an incorrect answer, or no answer^68^.

**Calculation abilities** were measured using the *Mental Calculation subtest* from the ZAREKI-R battery^68^ and the *Written Calculation subtest* from the WJ-III battery^69^. The *Mental Calculation subtest* includes eight additions, eight subtractions, and six multiplications presented orally by the examiner, with children having to respond verbally and without time constraints. Problems comprised both single- and double-digit operations. Scoring mirrored that of the *Word Problems subtest*: two points were awarded for a correct response without requesting a repetition; one point for a correct response after the examiner repeated the problem once; and zero points for an incorrect answer or no answer at all. In the *Written Calculation subtest*, children were asked to solve a list of increasingly difficult written calculation-problems, including single-, double-, and triple-digit operations. There was no time limit and the subtest was discontinued after six consecutive mistakes. Scores from the *Mental and Written Calculation subtests* were used to construct a latent variable^70^ representing overall calculation abilities.

**Math fluency abilities** were assessed using the *Math Fluency subtest* from the WJ-III battery^69^. In this timed written test, children were required to complete as many single-digit addition, subtraction, and multiplication calculations as possible within a 3-minute time-period. The score was calculated as the number of correct calculations completed within the allotted time, out of a maximum of 160.

#### Domain-general cognitive skills

The assessment of domain-general cognitive skills included evaluations of working memory, inhibition, cognitive flexibility, and selective attention skills.

**Working memory** was assessed using two distinct tests: the *Digit Span test* from the WISC-V^71^ for the verbal component; and the *Spatial Span test* from the Wechsler Nonverbal Scale of Ability^72^ for the visuo-spatial component. In the *Digit Span test*, children were asked to repeat progressively longer sequences of digits presented orally by the examiner in three conditions: (i) same order (“forward” condition); (ii) reverse order (“backward” condition); and (iii) ascending numerical order (“ascending” condition). For the *Spatial Span test*, a 3D board with 10 blocks was used, and children were asked to replicate sequences of block-touches either (i) in the same order (“forward” condition); or (ii) in reverse order (“backward” condition). For both tests, each condition began with two practice trials and was discontinued after two consecutive errors at a given sequence length (e.g., three digits or three touches). Accuracy scores from conditions requiring both storage and manipulation of information (i.e., backward digit span, ascending digit span, and backward spatial span)^73^ were taken into account to derive a latent working memory factor^70^.

**Inhibition and cognitive flexibility** skills were measured using the *Inhibition subtest* from the NEPSY-II battery^48^. Children were presented with a paper sheet displaying black-and-white stimuli arranged in rows (squares and circles in the first part, arrows in the second). This subtest included three conditions. The first condition, assessing naming speed, required children to name the stimuli as quickly as possible, regardless of their color. The second condition, targeting inhibition skills, asked children to provide the opposite name-label for each stimulus (e.g., say “square” for a circle, and “circle” for a square). The third condition assessed cognitive flexibility by having children name stimuli correctly when they were colored in black, and provide the opposite name-label when they were colored in white. A practice row containing a total of eight stimuli preceded each condition to ensure that the child understood the instructions. For each condition, the number of errors and response times were collected, and corrected-time scores were computed by adding, for each error, twice the participant’s average stimulus naming time to the total time^39^. To control for variability in naming speed, inhibition and flexibility scores were derived, as per guidelines^39^, by subtracting the corrected-time score of the naming condition from those of the inhibition and flexibility conditions, respectively.

**Selective attention** was evaluated using the *Cancellation subtest* from the WISC-V^71^. This subtest involved identifying animals among distractor-objects on an A3 paper sheet. The first condition featured a disorganized layout of stimuli, while the second condition used an orderly grid. Each condition allowed 45 s for children to cross out as many animals as possible. Training on a smaller A4 sheet preceded the actual test to ensure children understood the instructions. As both conditions assess the same selective attention process under varying levels of visual organization, the score was computed by summing the number of correctly identified animals across both conditions and subtracting errors^71^.

#### Domain-specific cognitive skills

The assessment of domain-specific cognitive skills included evaluations of symbolic as well as non-symbolic abilities. Symbolic skills were assessed using the following tasks: (i) transcoding, (ii) symbolic comparison, and (iii) number ordering; while non-symbolic skills were assessed through non-symbolic comparison tests involving (i) small (< 10) and (ii) large (> 10) quantities of dot-arrays.

**Transcoding skills** were evaluated using the *Number Dictation* and *Number Reading subtests* from the ZAREKI-R battery^68^. In the *Number Dictation subtest*, children were required to write down numbers spoken aloud by the examiner. Correct responses received two points if the number was accurately written without having to be repeated by the examiner; one point if correctly written after one repetition; and zero points for incorrect answers. This subtest consisted of a total of eight items and included one practice trial. The *Number Reading subtest* involved children reading aloud numbers displayed in symbolic digit format in the center of a paper sheet (one number per sheet). The scoring system awarded two points for correct responses on the first attempt; one point for self-corrected correct responses; and zero points for incorrect responses. This subtest also featured a total of eight items and one practice trial. Scores on both subtests were used to create a latent variable^70^ representing transcoding abilities.

**Symbolic comparison** skills were measured using the *Number Comparison subtest* from the Numeracy Screener battery^74,75^. The *Number Comparison subtest* consists of a total of 56 pairs of single-digit Arabic numerals ranging from 1 to 9, displayed on A4 paper sheets. Children were instructed to cross out the larger number (in numerosity) from each pair and had 1 min to complete as many pairs as possible. Before the actual test, children were trained on 12 practice pairs to get acquainted with the task, as per administration guidelines^74,75^. The total number of correct responses was recorded and entered into the analyses.

**Number ordering** skills were assessed using a test added later to the same Numeracy Screener battery^74,76^. This test presented children with a total of 48 triplets of single-digit Arabic numerals, ranging from 1 to 9, displayed on A4 paper sheets. Children had to determine whether the numbers were in ascending order or not and mark their response by crossing a check mark-shaped symbol for ordered triplets (e.g., 2 – 4 – 6) or a cross-shaped symbol for unordered triplets (e.g., 4 – 2 – 6). Children were given 1 min to complete as many triplets as possible. The test included four practice triplets to familiarize children with the task, as per administration guidelines^76^. The number of correct responses was recorded and entered into the analyses. Please note that this test was introduced into the current study-protocol shortly after data collection began, resulting in 19% of missing data for this variable (i.e., *N* = 88 children).

**Non-symbolic comparison of small quantities** skills were evaluated using the non-symbolic version of the symbolic comparison test from the Numeracy Screener^74,75^. In this test, a total of 56 pairs of dot-arrays, each containing between 1 and 9 dots, were presented on A4 paper sheets, with dot stimuli controlled for area and density^74^. Children were instructed to cross out the array with more dots as quickly and as accurately as possible, without counting. The test allowed a maximum of 1 min for completion and was preceded by 12 practice pairs to familiarize children with the task, as per administration guidelines^74,75^. The number of correct responses was recorded and entered into the analyses.

**Non-symbolic comparison of large quantities** skills were assessed by the Panamath software (version 1.22)^77^. The task began with the display of a white fixation-cross at the center of a grey computer screen, followed by the simultaneous presentation of two dot-arrays – one containing blue dots and the other yellow dots – on either side of the screen for 1506 milliseconds. Each dot-array consisted of numerosities ranging from 5 to 21 dots. Children were required to determine whether there were more blue or yellow dots by clicking the corresponding colored buttons on the keyboard (i.e., the *J* and *F* keys, respectively). Once a response was provided by the child, the examiner pressed the space bar to move on to the next trial. This is consistent with standard administration procedures of the Panamath software, ensuring that each child has sufficient time to respond^78,79^. The task included a total of 112 trials presented equally across four possible ratios (1.22; 1.37; 1.60; 2.60). The main task was preceded by two practice trials to help children understand the procedure and response-dynamics on the keyboard. ANS acuity was assessed using the Weber fraction (*w*), with lower values indicating more precise quantity representation^77^, and this measure was entered in the analyses.

#### Covariates

**Sex** was coded as − 0.5 for boys and 0.5 for girls.

**Age** was recorded in months.

**Testing month** was assigned numeric values ranging from 1 (September) to 11 (July).

**Nonverbal IQ** was assessed using the score at the *Matrices* subtest of the WISC-V^71^.

**SES** data were collected through questionnaires in which both parents reported their highest educational attainment and their occupation – two indicators consistently used to represent the SES construct^80,81^. Educational attainment was converted into years of education, ranging from 0 (no degree) to 20 (e.g., PhD degree). Occupations were categorized using ISCO-08 codes^82^ that were mapped into ISEI-08 scores^83^ through the R package *occupar* ^84^. The highest educational level and ISEI score of either parent were retained as indicators of parental education and occupation, respectively^8,80^. When information was available for only one parent, that information was used. In our sample, 37 participants (8%) had missing values for parental education only, 7 (1%) for parental occupation only and 51 (11%) for both indicators. When data were available for only one indicator (i.e., either education or occupation), the missing value was imputed using linear regression based on the other available variable. A principal component analysis (PCA) was then performed on the two SES indicators, and the first component – explaining 85% of the total variance – was extracted and the factor loadings were used as the SES composite score, consistent with prior approaches^8,85^.

**General anxiety** was assessed using the French version^86^ of the Revised Children’s Manifest Anxiety Scale (R-CMAS)^87^, a self-report questionnaire designed for children and adolescents aged 6 to 19. The R-CMAS comprises 37 dichotomous items (*Yes* = 1, *No* = 0), including 28 items measuring anxiety symptoms and 9 items forming a ‘Lie subscale’ designed to detect social desirability bias. The 28 anxiety items are distributed across three subscales – Physiological Anxiety (10 items), Worry/Oversensitivity (11 items), and Social Concerns/Concentration (7 items) – which jointly contribute to a total anxiety score. This total anxiety score, calculated by summing the responses to the 28 anxiety items, was entered in the analyses. Internal consistency for the total anxiety scale was good in the present sample (Cronbach’s α = 0.85).

### Data analyses

Data were analyzed using the R software (version 4.2.1)^88^. Structural equation modeling (SEM) was performed with the *lavaan* package^89^ to investigate the mediating effects of cognitive skills on the relationship between MA and mathematical outcomes. All variables were standardized prior to their inclusion in the SEM model to facilitate the interpretation of path coefficients. The Robust Maximum Likelihood (MLR) estimator was employed to compute robust standard errors and to account for nonnormality, while missing data were handled via the Full Information Maximum Likelihood (FIML) method. Model fit was evaluated using the χ² statistic, the Comparative Fit Index (CFI), the Root Mean Square Error of Approximation (RMSEA), and the Standardized Root Mean Square Residual (SRMR), with acceptable thresholds defined as CFI > 0.95, RMSEA < 0.08, and SRMR < 0.10^41^. The analysis proceeded in two stages: first, a confirmatory factor analysis (CFA) was conducted to evaluate the measurement model and confirm that the latent variables adequately represented their observed indicators; second, the SEM mediation model – which examined the relationships between MA and mathematical outcomes through both domain-general and domain-specific cognitive factors – was tested (see Fig. S1 in the Supplementary Information). Percentile bootstrap confidence intervals (95% CIs) with 5000 iterations were computed to evaluate indirect effects^90^. Indirect effects were deemed significant if their CI did not include 0^90^. Mediation effect sizes were reported through both the type of mediation (full or partial) and the magnitude of the indirect effects, as indicated by the standardized indirect effect coefficients^42^.

## Supplementary Information

Below is the link to the electronic supplementary material.

Supplementary Material 1

## Data Availability

The datasets generated and analyzed during the present study are available from the corresponding authors upon request. Analysis code is available on OSF at: https://osf.io/uzrey/?view_only=986654f41a1d4ca3995a2dff6e4df8e5.
