# Supplementary material for: Domain-general and domain-specific cognitive factors mediating the relationship between math anxiety and mathematical performance in primary school children
Source: Sci Rep. 2026 Jan 14;16:1987. doi: 10.1038/s41598-025-30898-2 (PMC12808763; doi:10.1038/s41598-025-30898-2)
Supplement: Supplementary file 1 — Supplementary Material 1 [file 41598_2025_30898_MOESM1_ESM.pdf]

**Supplementary Information**

# Domain-general and domain-specific cognitive factors mediating the relationship between math anxiety and mathematical performance in primary school children

This PDF file includes:

- Fig. S1
- Tables S1 to S3

**Fig. S1. SEM mediation model tested to investigate the cognitive mediators of the relationship between MA and mathematical outcomes.** Latent variables are depicted by ovals and observed variables by squares. Domain-general factors are represented in purple and domain-specific factors in red. Covariates were regressed onto each mediator and dependent variable. Covariances between all mediators were specified in the model, but for clarity are not represented on the present figure. ANS = approximate number system; SES = socioeconomic status.

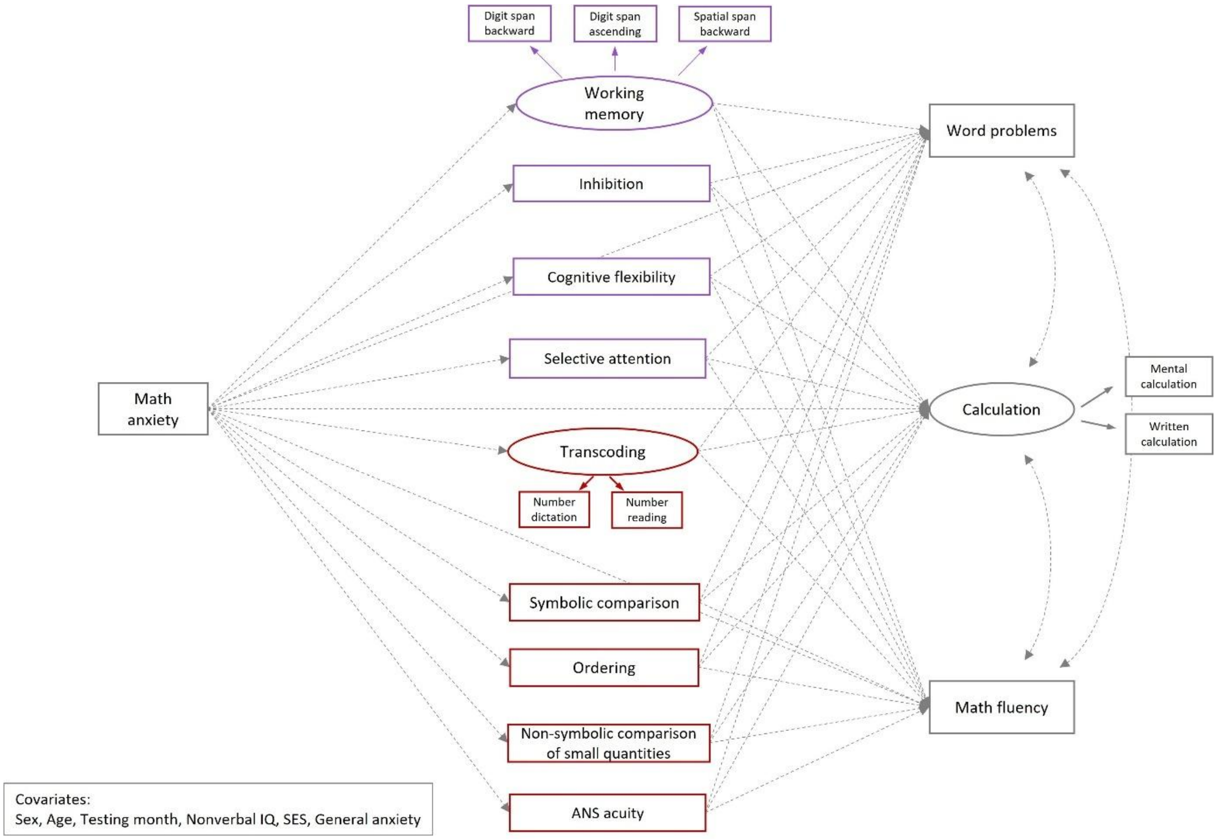

**Table S1**. **Bivariate correlations among observed variables included in the SEM model.** Zero-order correlations are provided. Sex was dummy coded with boys = –0.5 and girls = 0.5. ANS = approximate number system. SES = socioeconomic status. **p* < .05.

| | 1 | 2 | 3 | 4 | 5 | 6 | 7 | 8 | 9 | 10 | 11 | 12 | 13 | 14 | 15 | 16 | 17 | 18 | 19 | 20 | 21 | 22 | 23 |
|---|---|---|---|---|---|---|---|---|---|---|---|---|---|---|---|---|---|---|---|---|---|---|---|
| 1. Math anxiety | _ | | | | | | | | | | | | | | | | | | | | | | |
| 2. Word problems | −.30* | _ | | | | | | | | | | | | | | | | | | | | | |
| 3. Mental calculation | −.35* | .62* | _ | | | | | | | | | | | | | | | | | | | | |
| 4. Written calculation | −.30* | .55* | .67* | _ | | | | | | | | | | | | | | | | | | | |
| 5. Math fluency | −.30* | .51* | .60* | .67* | _ | | | | | | | | | | | | | | | | | | |
| 6. Digit span backward | −.17* | .37* | .34* | .37* | .32* | _ | | | | | | | | | | | | | | | | | |
| 7. Digit span ascending | −.22* | .49* | .41* | .38* | .37* | .34* | _ | | | | | | | | | | | | | | | | |
| 8. Spatial span backward | −.22* | .40* | .41* | .35* | .27* | .36* | .30* | _ | | | | | | | | | | | | | | | |
| 9. Inhibition | .05 | −.24* | −.25* | −.28* | −.23* | −.21* | −.19* | −.27* | _ | | | | | | | | | | | | | | |
| 10. Flexibility | .21* | −.32* | −.37* | −.31* | −.34* | −.25* | −.23* | −.20* | .35* | _ | | | | | | | | | | | | | |
| 11. Selective attention | −.09 | .20* | .19* | .22* | .32* | .18* | .15* | .19* | −.20* | −.25* | _ | | | | | | | | | | | | |
| 12. Number dictation | −.31* | .45* | .50* | .42* | .44* | .25* | .35* | .27* | −.23* | −.29* | .11* | _ | | | | | | | | | | | |
| 13. Number reading | −.26* | .41* | .47* | .39* | .37* | .22* | .30* | .25* | −.27* | −.30* | .12* | .55* | _ | | | | | | | | | | |
| 14. Symbolic comparison | −.19* | .34* | .37* | .38* | .54* | .22* | .28* | .21* | −.26* | −.32* | .40* | .28* | .16* | _ | | | | | | | | | |
| 15. Ordering | −.27* | .44* | .42* | .42* | .59* | .32* | .35* | .33* | −.31* | −.39* | .35* | .26* | .24* | .56* | _ | | | | | | | | |
| 16. Non-symbolic comparison of small quantities | −.07 | .23* | .26* | .26* | .36* | .12* | .16* | .15* | −.20* | −.24* | .35* | .15* | .05 | .76* | .43* | _ | | | | | | | |
| 17. ANS acuity | .09 | −.16* | −.16* | −.23* | −.15* | −.15* | −.15* | −.12* | .13* | .14* | −.11* | −.13* | −.14* | −.15* | −.17* | −.10* | _ | | | | | | |
| 18. Sex | .12* | −.13* | −.16* | −.10* | −.18* | −.05 | −.11* | −.07 | .04 | .18* | .03 | −.10* | −.12* | −.04 | −.02 | .01 | −.13* | _ | | | | | |
| 19. Age | −.07 | .15* | .17* | .25* | .21* | .08 | .07 | .06 | −.04 | −.09 | .21* | .06 | .10* | .22* | .24* | .24* | −.06 | .00 | _ | | | | |
| 20. Testing month | −.01 | .15* | .12* | .25* | .22* | .07 | .06 | .00 | .03 | −.05 | .16* | .07 | .08 | .16* | .22* | .15* | −.13* | .07 | .68* | _ | | | |
| 21. Nonverbal IQ | −.21* | .32* | .29* | .29* | .17* | .21* | .18* | .23* | −.18* | −.21* | .24* | .21* | .19* | .14* | .19* | .14* | −.17* | .14* | .17* | .14* | _ | | |
| 22. SES composite | −.18* | .38* | .35* | .28* | .14* | .26* | .26* | .28* | −.26* | −.15* | .13* | .24* | .24* | .13* | .12* | .06 | −.07 | −.01 | −.02 | −.01 | .31* | _ | |
| 23. General anxiety | .41* | −.15* | −.13* | −.11* | −.10* | −.16* | −.17* | −.11* | .01 | .15* | −.04 | −.16* | −.13* | −.11* | −.13* | −.06 | −.04 | .21* | −.04 | .08 | −.11* | −.14* | _ |

**Table S2. Complete SEM model with all parameter estimates.** Both standardized (β) and unstandardized (*b*) estimates are reported. SE = standard error; MA = math anxiety; ANS = approximate number system; SES = socioeconomic status.

**A. Paths from MA and covariates to cognitive variables.**

| **Paths** | **β** | ***b*** | **SE** | ***p*** |
|---|---|---|---|---|
| MA → Working memory | –0.18 | –0.22 | 0.08 | .005 |
| MA → Inhibition | –0.01 | –0.01 | 0.05 | .853 |
| MA → Flexibility | 0.12 | 0.12 | 0.06 | .030 |
| MA → Selective attention | –0.03 | –0.03 | 0.05 | .576 |
| MA → Transcoding | –0.29 | –0.33 | 0.08 | < .001 |
| MA → Symbolic comparison | –0.14 | –0.14 | 0.05 | .007 |
| MA → Ordering | –0.22 | –0.22 | 0.05 | < .001 |
| MA → Non-symbolic comparison of small quantities | –0.02 | –0.02 | 0.05 | .711 |
| MA → ANS acuity | 0.10 | 0.11 | 0.06 | .062 |
| Sex → Working memory | –0.11 | –0.28 | 0.14 | .047 |
| Sex → Inhibition | 0.05 | 0.11 | 0.09 | .225 |
| Sex → Flexibility | 0.18 | 0.37 | 0.09 | < .001 |
| Sex → Selective attention | 0.01 | 0.02 | 0.09 | .796 |
| Sex → Transcoding | –0.13 | –0.31 | 0.12 | .008 |
| Sex → Symbolic comparison | –0.02 | –0.05 | 0.09 | .602 |
| Sex → Ordering | –0.03 | –0.06 | 0.10 | .557 |
| Sex → Non-symbolic comparison of small quantities | 0.01 | 0.02 | 0.09 | .828 |
| Sex → ANS acuity | –0.09 | –0.19 | 0.08 | .024 |
| Age → Working memory | 0.06 | 0.07 | 0.10 | .434 |
| Age → Inhibition | –0.11 | –0.11 | 0.06 | .072 |
| Age → Flexibility | –0.04 | –0.04 | 0.06 | .512 |
| Age → Selective attention | 0.18 | 0.18 | 0.06 | .001 |

| | | | | |
|---|---|---|---|---|
| Age → Transcoding | –0.00 | –0.00 | 0.07 | .956 |
| Age → Symbolic comparison | 0.18 | 0.18 | 0.06 | .005 |
| Age → Ordering | 0.11 | 0.11 | 0.07 | .080 |
| Age → Non-symbolic comparison of small quantities | 0.25 | 0.25 | 0.06 | < .001 |
| Age → ANS acuity | 0.07 | 0.07 | 0.08 | .376 |
| Testing month → Working memory | 0.02 | 0.03 | 0.09 | .791 |
| Testing month → Inhibition | 0.11 | 0.11 | 0.06 | .070 |
| Testing month → Flexibility | –0.02 | –0.02 | 0.06 | .790 |
| Testing month → Selective attention | 0.01 | 0.01 | 0.06 | .904 |
| Testing month → Transcoding | 0.09 | 0.11 | 0.08 | .184 |
| Testing month → Symbolic comparison | 0.05 | 0.05 | 0.06 | .438 |
| Testing month → Ordering | 0.15 | 0.15 | 0.07 | .020 |
| Testing month → Non-symbolic comparison of small quantities | –0.02 | –0.02 | 0.06 | .741 |
| Testing month → ANS acuity | –0.15 | –0.15 | 0.09 | .074 |
| Nonverbal IQ → Working memory | 0.20 | 0.25 | 0.07 | .001 |
| Nonverbal IQ → Inhibition | –0.12 | –0.12 | 0.05 | .007 |
| Nonverbal IQ → Flexibility | –0.17 | –0.17 | 0.04 | < .001 |
| Nonverbal IQ → Selective attention | 0.17 | 0.17 | 0.05 | < .001 |
| Nonverbal IQ → Transcoding | 0.15 | 0.17 | 0.07 | .012 |
| Nonverbal IQ → Symbolic comparison | 0.05 | 0.05 | 0.05 | .249 |
| Nonverbal IQ → Ordering | 0.09 | 0.09 | 0.05 | .089 |
| Nonverbal IQ → Non-symbolic comparison of small quantities | 0.09 | 0.09 | 0.05 | .059 |
| Nonverbal IQ → ANS acuity | –0.13 | –0.13 | 0.06 | .039 |
| SES → Working memory | 0.34 | 0.42 | 0.09 | < .001 |
| SES → Inhibition | –0.24 | –0.24 | 0.05 | < .001 |
| SES → Flexibility | –0.08 | –0.08 | 0.05 | .090 |

| | | | | |
|---|---|---|---|---|
| SES → Selective attention | 0.09 | 0.09 | 0.05 | .094 |
| SES → Transcoding | 0.23 | 0.26 | 0.06 | < .001 |
| SES → Symbolic comparison | 0.09 | 0.09 | 0.05 | .085 |
| SES → Ordering | 0.06 | 0.06 | 0.05 | .269 |
| SES → Non-symbolic comparison of small quantities | 0.03 | 0.03 | 0.05 | .511 |
| SES → ANS acuity | –0.02 | –0.02 | 0.05 | .666 |
| General anxiety → Working memory | –0.09 | –0.11 | 0.08 | .161 |
| General anxiety → Inhibition | –0.05 | –0.05 | 0.05 | .278 |
| General anxiety → Flexibility | 0.04 | 0.04 | 0.06 | .556 |
| General anxiety → Selective attention | 0.01 | 0.01 | 0.05 | .925 |
| General anxiety → Transcoding | –0.02 | –0.02 | 0.07 | .797 |
| General anxiety → Symbolic comparison | –0.03 | –0.03 | 0.05 | .562 |
| General anxiety → Ordering | –0.02 | –0.02 | 0.05 | .749 |
| General anxiety → Non-symbolic comparison of small quantities | –0.03 | –0.03 | 0.05 | .484 |
| General anxiety → ANS acuity | –0.07 | –0.07 | 0.05 | .164 |

**B. Paths from cognitive variables and covariates to mathematics outcomes.**

| Paths | Word problems | | | | Calculation | | | | Math fluency | | | |
|---|---|---|---|---|---|---|---|---|---|---|---|---|
| | **β** | ***b*** | **SE** | ***p*** | **β** | ***b*** | **SE** | ***p*** | **β** | ***b*** | **SE** | ***p*** |
| MA → Math (total effect c) | –0.19 | –0.19 | 0.05 | < .001 | –0.30 | –0.65 | 0.13 | < .001 | –0.26 | –0.26 | 0.05 | < .001 |
| MA → Math (direct effect c’) | –0.03 | –0.03 | 0.04 | .442 | –0.10 | –0.22 | 0.10 | .022 | –0.06 | –0.06 | 0.04 | .139 |
| Working memory → Math | 0.62 | 0.50 | 0.10 | < .001 | 0.49 | 0.87 | 0.26 | .001 | 0.17 | 0.14 | 0.08 | .073 |
| Inhibition → Math | 0.09 | 0.09 | 0.04 | .051 | 0.04 | 0.09 | 0.10 | .337 | 0.05 | 0.05 | 0.04 | .208 |
| Flexibility → Math | –0.01 | –0.01 | 0.06 | .869 | –0.04 | –0.07 | 0.10 | .449 | 0.03 | 0.03 | 0.04 | .504 |
| Selective attention → Math | –0.05 | –0.05 | 0.04 | .222 | –0.05 | –0.10 | 0.11 | .326 | 0.06 | 0.06 | 0.04 | .148 |
| Transcoding → Math | 0.16 | 0.14 | 0.07 | .064 | 0.32 | 0.59 | 0.18 | .001 | 0.29 | 0.25 | 0.06 | < .001 |

| | | | | | | | | | | | | |
|---|---|---|---|---|---|---|---|---|---|---|---|---|
| Symbolic comparison → Math | –0.02 | –0.02 | 0.06 | .770 | 0.04 | 0.09 | 0.16 | .591 | 0.25 | 0.25 | 0.06 | < .001 |
| Ordering → Math | 0.02 | 0.02 | 0.08 | .822 | 0.03 | 0.06 | 0.17 | .735 | 0.27 | 0.27 | 0.07 | < .001 |
| Non-symbolic comparison of small quantities → Math | 0.08 | 0.08 | 0.06 | .137 | 0.10 | 0.20 | 0.13 | .128 | –0.02 | –0.02 | 0.05 | .659 |
| ANS acuity → Math | 0.03 | 0.03 | 0.04 | .404 | –0.01 | –0.03 | 0.10 | .751 | 0.02 | 0.02 | 0.03 | .493 |
| Sex → Math | –0.04 | –0.09 | 0.08 | .313 | –0.07 | –0.29 | 0.19 | .117 | –0.11 | –0.23 | 0.07 | .002 |
| Age → Math | 0.01 | 0.01 | 0.05 | .931 | 0.09 | 0.20 | 0.12 | .097 | –0.03 | –0.03 | 0.05 | .508 |
| Testing month → Math | 0.07 | 0.07 | 0.06 | .226 | 0.06 | 0.13 | 0.12 | .317 | 0.10 | 0.10 | 0.05 | .047 |
| Nonverbal IQ → Math | 0.05 | 0.05 | 0.05 | .233 | 0.05 | 0.10 | 0.10 | .304 | –0.02 | –0.02 | 0.04 | .652 |
| SES → Math | 0.06 | 0.06 | 0.05 | .230 | 0.04 | 0.08 | 0.12 | .515 | –0.09 | –0.09 | 0.05 | .054 |
| General anxiety → Math | 0.08 | 0.08 | 0.04 | .051 | 0.13 | 0.27 | 0.10 | .008 | 0.09 | 0.09 | 0.04 | .017 |

**C. Variances.**

| **Variances** | **β** | ***b*** | **SE** | ***p*** |
|---|---|---|---|---|
| MA | 1.00 | 1.00 | 0.07 | < .001 |
| Working memory | 0.66 | 1.00 | – | – |
| Inhibition | 0.91 | 0.91 | 0.09 | < .001 |
| Flexibility | 0.89 | 0.88 | 0.15 | < .001 |
| Selective attention | 0.90 | 0.90 | 0.06 | < .001 |
| Transcoding | 0.74 | 1.00 | – | – |
| Symbolic comparison | 0.90 | 0.91 | 0.06 | < .001 |
| Ordering | 0.85 | 0.85 | 0.07 | < .001 |
| Non-symbolic comparison of small quantities | 0.92 | 0.93 | 0.07 | < .001 |
| ANS acuity | 0.94 | 0.94 | 0.32 | .003 |
| Word problems | 0.41 | 0.41 | 0.05 | < .001 |
| Calculation | 0.22 | 1.00 | – | – |
| Math fluency | 0.43 | 0.43 | 0.04 | < .001 |
| Sex | 1.00 | 0.25 | 0.00 | < .001 |
| Age | 1.00 | 1.00 | 0.05 | < .001 |
| Testing month | 1.00 | 1.00 | 0.03 | < .001 |
| Nonverbal IQ | 1.00 | 1.00 | 0.06 | < .001 |

| | | | | |
|---|---|---|---|---|
| SES | 1.00 | 1.00 | 0.06 | < .001 |
| General anxiety | 1.00 | 1.00 | 0.06 | < .001 |

**D. Covariances.**

| **Covariances** | **β** | ***b*** | **SE** | ***p*** |
|---|---|---|---|---|
| Working memory ↔ Inhibition | –0.31 | –0.30 | 0.06 | < .001 |
| Working memory ↔ Flexibility | –0.28 | –0.27 | 0.06 | < .001 |
| Working memory ↔ Selective attention | 0.22 | 0.21 | 0.07 | .002 |
| Working memory ↔ Transcoding | 0.52 | 0.52 | 0.07 | < .001 |
| Working memory ↔ Symbolic comparison | 0.34 | 0.32 | 0.06 | < .001 |
| Working memory ↔ Ordering | 0.55 | 0.51 | 0.07 | < .001 |
| Working memory ↔ Non-symbolic comparison of small quantities | 0.19 | 0.19 | 0.06 | .003 |
| Working memory ↔ ANS acuity | –0.23 | –0.23 | 0.05 | < .001 |
| Inhibition ↔ Flexibility | 0.32 | 0.28 | 0.06 | < .001 |
| Inhibition ↔ Selective attention | –0.15 | –0.14 | 0.04 | .001 |
| Inhibition ↔ Transcoding | –0.27 | –0.26 | 0.06 | < .001 |
| Inhibition ↔ Symbolic comparison | –0.23 | –0.21 | 0.05 | < .001 |
| Inhibition ↔ Ordering | –0.30 | –0.26 | 0.05 | < .001 |
| Inhibition ↔ Non-symbolic comparison of small quantities | –0.18 | –0.16 | 0.04 | < .001 |
| Inhibition ↔ ANS acuity | 0.12 | 0.11 | 0.03 | .001 |
| Flexibility ↔ Selective attention | –0.21 | –0.19 | 0.05 | < .001 |
| Flexibility ↔ Transcoding | –0.29 | –0.28 | 0.07 | < .001 |
| Flexibility ↔ Symbolic comparison | –0.27 | –0.24 | 0.05 | < .001 |
| Flexibility ↔ Ordering | –0.34 | –0.30 | 0.05 | < .001 |
| Flexibility ↔ Non-symbolic comparison of small quantities | –0.22 | –0.20 | 0.05 | < .001 |
| Flexibility ↔ ANS acuity | 0.14 | 0.13 | 0.04 | < .001 |
| Selective attention ↔ Transcoding | 0.07 | 0.07 | 0.05 | .198 |
| Selective attention ↔ Symbolic comparison | 0.36 | 0.32 | 0.05 | < .001 |
| Selective attention ↔ Ordering | 0.30 | 0.26 | 0.05 | < .001 |

| | | | | |
|---|---|---|---|---|
| Selective attention ↔ Non-symbolic comparison of small quantities | 0.30 | 0.28 | 0.05 | < .001 |
| Selective attention ↔ ANS acuity | –0.07 | –0.07 | 0.04 | .059 |
| Transcoding ↔ Symbolic comparison | 0.22 | 0.20 | 0.06 | < .001 |
| Transcoding ↔ Ordering | 0.21 | 0.20 | 0.07 | .003 |
| Transcoding ↔ Non-symbolic comparison of small quantities | 0.09 | 0.09 | 0.06 | .149 |
| Transcoding ↔ ANS acuity | –0.15 | –0.15 | 0.05 | .006 |
| Symbolic comparison ↔ Ordering | 0.50 | 0.44 | 0.05 | < .001 |
| Symbolic comparison ↔ Non-symbolic comparison of small quantities | 0.75 | 0.69 | 0.06 | < .001 |
| Symbolic comparison ↔ ANS acuity | –0.13 | –0.12 | 0.03 | < .001 |
| Ordering ↔ Non-symbolic comparison of small quantities | 0.38 | 0.34 | 0.05 | < .001 |
| Ordering ↔ ANS acuity | –0.15 | –0.13 | 0.04 | .001 |
| Non-symbolic comparison of small quantities ↔ ANS acuity | –0.09 | –0.08 | 0.04 | .020 |
| Word problems ↔ Calculation | 0.20 | 0.13 | 0.07 | .058 |
| Word problems ↔ Math fluency | 0.10 | 0.04 | 0.03 | .125 |
| Calculation ↔ Math fluency | 0.63 | 0.41 | 0.06 | < .001 |

**Table S3. SEMA questionnaire translated in French.**

| | |
|---|---|
| Instructions: « Je vais te lire quelques questions de mathématiques. Pour chaque question, je veux que tu t'imagines en train d'y répondre. Ensuite, montre-moi comment tu te sens à l'idée de répondre à cette question. Par exemple, tu peux te sentir *(montrer en même temps)* : Pas du tout nerveux, Un peu nerveux, Moyennement nerveux, Très nerveux ou Très très neveux. Attention, tu ne dois pas réellement répondre à la question, tu dois juste t'imaginer en train de répondre et me montrer comment tu te sens. Voici un exemple. » | |
| Exemple 1. | Qui est le président de la France ? |
| Item 1. | Thomas a acheté 2 pizzas de 6 parts chacune. Combien de parts au total Thomas a-t-il dû partager avec ses amis ? |
| Item 2. | L'opération suivante est-elle juste : 9 + 7 = 18 ? |
| Item 3. | Combien d'argent a Anne si elle possède 2 pièces de 1 euro et 4 pièces de 1 centime ? |
| Item 4. | Comment écris-tu le nombre *quatre cent quatre-vingt-deux* ? |
| Item 5. | Dessine-moi les aiguilles d'une horloge pour qu'on puisse lire 15h15. |
| Item 6. | Dessine-moi un carré et un triangle au tableau. |
| Item 7. | Compte de 5 en 5 à partir de 10 jusqu'à 55. |
| Item 8. | Quelle heure sera-t-il dans 20 minutes ? |
| Item 9. | L'opération suivante est-elle juste : 15 – 7 = 8 ? |
| Item 10. | Léa a plus d'argent que Marie. Marie a plus d'argent que Julie. Qui a le plus d'argent entre Léa et Julie ? |
| Instructions : « Maintenant, je vais te montrer des phrases sur des situations qui impliquent les maths. Pour chaque phrase, imagine-toi que la situation est en train d'arriver et montre-moi comment tu te sens, comme on vient de faire. Voici un exemple. » | |
| Exemple 2. | Tu es sur le point de faire un tour de montagnes russes. |
| Item 11. | Tu es en classe en train de faire des maths et ton professeur est sur le point d'enseigner quelque chose de nouveau. |
| Item 12. | Tu dois t'asseoir pour commencer tes devoirs de maths. |
| Item 13. | Tu comptes tout l'argent que tu as dans ta tirelire. |
| Item 14. | Quelqu'un te demande de couper une tarte aux pommes en quatre parts égales. |
| Item 15. | Tu es sur le point de faire une évaluation de maths. |

| | |
|---|---|
| Item 16. | Tu ne comprends pas quelque chose en maths. Tu demandes à ton professeur de t'aider. |
| Item 17. | Ton professeur te donne un tas de problèmes d'addition à résoudre. |
| Item 18. | Ton professeur te donne un tas de problèmes de soustraction à résoudre. |
| Item 19. | Tu es en classe en train de résoudre un problème de maths au tableau. |
| Item 20. | Tu es en train d'écouter ton professeur qui t'explique comment résoudre un problème de maths. |
